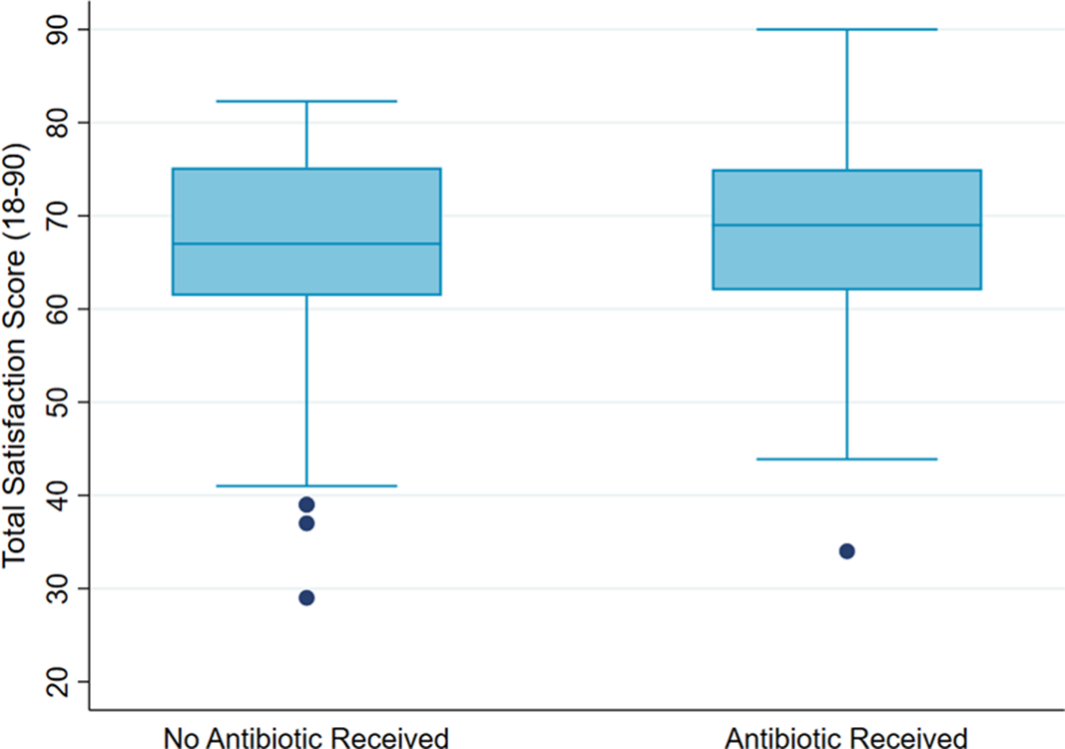# Veteran Satisfaction for Upper Respiratory-Tract Infection (URI) Visits Is Not Associated with Antibiotic Receipt But Is Associated with Antibiotic Expectation

**DOI:** 10.1017/ash.2021.61

**Published:** 2021-07-29

**Authors:** Milner Owens Staub, Rachael Pellegrino, Morgan Johnson, Erin Gettler, Christianne Roumie, Robert Dittus, Todd Hulgan

## Abstract

**Background:** Antibiotics are not recommended but are often prescribed for upper respiratory-tract infections (URIs). Prescribers cite patient expectation as a driver of inappropriate antibiotic prescribing; prior literature has demonstrated higher satisfaction scores in patients who receive antibiotics compared to those who do not. We assessed whether veteran satisfaction at URI visits was associated with antibiotic receipt or with reported expectation for antibiotics. **Methods:** We surveyed veterans with documented URI encounters in the Veterans’ Affairs Tennessee Valley Healthcare System between January 1, 2018, and December 31, 2019. Patients not evaluated in person, with documented dementia, or who died prior to the study start date were excluded. Veterans were asked to recall their URI visit and to complete the Patient Safety Questionnaire (PSQ)-18 (Rand Corporation) and questions assessing antibiotic expectations. The PSQ-18, an 18-item survey that assesses patient satisfaction, uses a 5-point Likert scale (ie, strongly disagree, disagree, uncertain, agree, strongly agree), yielding a composite score of 18–90. Higher scores represent more satisfaction with care. Demographic and visit-specific information were extracted via chart review. We used multivariable linear regression to assess differences in composite PSQ-18 satisfaction scores between those who did and did not receive an antibiotic, adjusted for patient and visit characteristics, and to assess differences in satisfaction scores for those who did and did not report expecting antibiotics, adjusted for antibiotic receipt. **Results:** We identified 1,435 patients seen for URI at 17 sites. After exclusions, 1,343 veterans were eligible for chart abstraction. After excluding 42 responders who responded after study close or returned blank surveys, the final analytic cohort included 432 (32.2%) of 1,343 responders; 225 (52.1%) received an antibiotic and 207 (47.9%) did not. Mean total satisfaction for veterans who received an antibiotic was 67.8 (SD, ±9.4) compared to 66.7 (SD, ±9.7) for those who did not (Figure 1). Increased total satisfaction was not significantly associated with antibiotic receipt (0.65; 95% CI, −2.0 to 3.3). Most veterans (72.0%) disagreed that visit satisfaction depended on antibiotic receipt. However, only 30.8% reported that they would not expect an antibiotic for URI visits. A significant reduction in total satisfaction (−4.1; 95% CI, −6.3 to −1.9) was associated with expecting compared to not expecting an antibiotic. **Conclusions:** Our findings suggest that prescribing an antibiotic is not associated with increased veteran satisfaction for URI visits but is associated with expecting an antibiotic. Future work will evaluate methods to change veteran antibiotic expectations.

**Funding:** No

**Disclosures:** None

**Figure 1.**